# Synovial fluid proteome changes in ACL injury-induced posttraumatic osteoarthritis: Proteomics analysis of porcine knee synovial fluid

**DOI:** 10.1371/journal.pone.0212662

**Published:** 2019-03-01

**Authors:** Ata M. Kiapour, Jakob T. Sieker, Benedikt L. Proffen, TuKiet T. Lam, Braden C. Fleming, Martha M. Murray

**Affiliations:** 1 Department of Orthopaedic Surgery, Boston Children's Hospital, Harvard Medical School, Boston, MA, United States of America; 2 Department of Molecular Biophysics and Biochemistry, Yale University, New Haven, CT, United States of America; 3 MS & Proteomics Resource, W.M. Keck Biotechnology Resource Laboratory, Yale University, New Haven, CT, United States of America; 4 Department of Orthopaedics, Warren Alpert Medical School of Brown University & Rhode Island Hospital, Providence, RI, United States of America; University of Umeå, SWEDEN

## Abstract

Surgical transection of the anterior cruciate ligament (ACL) in the porcine model leads to posttraumatic osteoarthritis if left untreated. However, a recently developed surgical treatment, bridge-enhanced ACL repair, prevents further cartilage damage. Since the synovial fluid bathes all the intrinsic structures of knee, we reasoned that a comparative analysis of synovial fluid protein contents could help to better understand the observed chondroprotective effects of the bridge-enhanced ACL repair. We hypothesized that post-surgical changes in the synovial fluid proteome would be different in the untreated and repaired knees, and those changes would correlate with the degree of cartilage damage. Thirty adolescent Yucatan mini-pigs underwent unilateral ACL transection and were randomly assigned to either no further treatment (ACLT, n = 14) or bridge-enhanced ACL repair (BEAR, n = 16). We used an isotopically labeled high resolution LC MS/MS-based proteomics approach to analyze the protein profile of synovial fluid at 6 and 12 months after ACL transection in untreated and repaired porcine knees. A linear mixed effect model was used to compare the normalized protein abundance levels between the groups at each time point. Bivariate linear regression analyses were used to assess the correlations between the macroscopic cartilage damage (total lesion area) and normalized abundance levels of each of the identified secreted proteins. There were no significant differences in cartilage lesion area or quantitative abundance levels of the secreted proteins between the ACLT and BEAR groups at 6 months. However, by 12 months, greater cartilage damage was seen in the ACLT group compared to the BEAR group (p = 0.005). This damage was accompanied by differences in the abundance levels of secreted proteins, with higher levels of Vitamin K-dependent protein C (p = 0.001), and lower levels of Apolipoprotein A4 (p = 0.021) and Cartilage intermediate layer protein 1 (p = 0.049) in the ACLT group compared to the BEAR group. There were also group differences in the secreted proteins that significantly changed in abundance between 6 and 12 months in ACLT and BEAR knees. Increased concentration of Ig lambda-1 chain C regions and decreased concentration of Hemopexin, Clusterin, Coagulation factor 12 and Cartilage intermediate layer protein 1 were associated with greater cartilage lesion area. In general, ACLT knees had higher concentrations of pro-inflammatory proteins and lower concentrations of anti-inflammatory proteins than BEAR group. In addition, the ACLT group had a lower and declining synovial concentrations of CILP, in contrast to a consistently high abundance of CILP in repaired knees. These differences suggest that the knees treated with bridge-enhanced ACL repair may be maintaining an environment that is more protective of the extracellular matrix, a function which is not seen in the ACLT knees.

## Introduction

Anterior cruciate ligament (ACL) injuries are has been linked to increased risk of posttraumatic osteoarthritis (OA) in humans and animal models [1–3]. Synovial fluid has been an attractive source to identify new biomarkers for monitoring joint health and a better understanding of the disease pathophysiology. This attraction is primarily due to the fact the synovial fluid bathes all the intrinsic structures of diarthrodial joints, including articular cartilage and synovium, both of which have shown to be actively involved in OA development [4, 5]. Additionally, alterations in these structures due to OA may be directly reflected in the composition of synovial fluid, which could be correlated to disease severity and progression. Recent advances in high-throughput and sensitive mass spectrometry (MS)-based approaches have facilitated protein profiling of complex biological fluids including synovial fluid. As such, this technology has emerged as a powerful and reproducible technique to identify proteins involved in disease etiology and pathogenesis, as well as potential biomarkers for a range of diseases, including arthritis [6–11].

Recently, a biologically augmented ACL repair procedure, bridge-enhanced ACL repair, has shown to be successful in reducing macroscopic evidence of posttraumatic OA following ACL injury in porcine knees [12]. This new surgical technique uses a combination of a novel extracellular matrix-based scaffold to augment a suture repair of the torn ACL [13]. Using the porcine ACL transection model, where the untreated knee progresses to posttraumatic OA and the repaired knee promotes cartilage preservation, allows us to compare these two different consequences of an identical surgical injury and potentially identify mechanisms and biomarkers for posttraumatic OA development.

Compared to other animal models, the porcine knee has been shown to be closest to the human based on its size, anatomy and functional dependency on the ACL [14]. Furthermore, porcine knee develops posttraumatic OA following ACL transection in a pattern similar to that reported in humans, but at a faster rate, with the joint changes at 1 year reflective of those seen at 10–15 years after ACL reconstruction in humans [12]. This faster onset of post-traumatic OA allows for more rapid assessment of factors that may influence the development of posttraumatic OA following ACL injury and treatment. The high degree of similarity between human and porcine synovial fluid [15]. further justifies the porcine knee as a suitable model to study the biology of OA.

In the current work, we used an isotopically labeled high resolution LC MS/MS-based proteomics approach to analyze the protein profile of synovial fluid at 6 and 12 months after ACL transection in untreated and repaired porcine knees. Our primary aim was to determine how the synovial fluid proteome differs between the two groups in an effort to identify candidate proteins that may be associated with the development of posttraumatic OA. We hypothesized that the development of macroscopic cartilage damage following surgical ACL transection would be accompanied by differential changes in synovial fluid proteome in untreated knees compared to repaired joints.

## Materials and methods

### Study design

This study was approved by the Institutional Animal Care and Use Committee at Boston Children's Hospital. The study was designed as a randomized controlled large animal trial with cross-sectional outcome assessments at two post-injury time points. The study was designed as a randomized controlled large animal trial with cross-sectional outcome assessments at two post-injury time points. A total of 30 adolescent Yucatan mini-pigs (ages 14–16 months) underwent unilateral ACL transection and were randomly treated with bridge-enhanced ACL repair (BEAR, n = 16) or left untreated (ACLT, n = 14). Animals were obtained from LoneStar Laboratory Swine (Sioux Center, IA, USA). A computer based random permutation of the complete set of animal identifiers was used to randomize treatment allocation and side of unilateral surgery. Half of the animals within each treatment group were euthanized at 6 months, while the other half was followed up to 12 months post-injury. The histological, biomechanical and OA-related outcomes for these animals have been previously reported by our group [12, 16]. For the current study, synovial fluid from an additional 6 healthy intact age-matched adolescent Yucatan minipigs was also used as a control.

### Surgical procedure

All animals were acclimated to the animal care facility environment for a minimum of 7 days prior to any surgical procedures. Animals allocated to ACLT or BEAR groups underwent ACL surgery under general anesthesia and postoperative housing as previously reported [12, 16]. In brief, the ACL was transected surgically and for the animals assigned to BEAR group, the torn ACL was repaired using an extracellular matrix–based scaffold soaked with autologous blood as previously described [17]. After surgery, all animals were housed in individualized pens and checked multiple times a day for 4 weeks. They were then shipped to a farm for long-term care (Coyote Consulting Corp Inc, Douglas, Massachusetts). Full weightbearing status was achieved within 48 to 72 hours and the animals were allowed ad libitum activity. Two animals (both in the ACLT group) developed subcutaneous abscesses near the jaw that were treated with short-term oral antibiotics. These animals were included in the study as there was no visual evidence of joint synovitis at the time of dissection. In the BEAR group, one animal (12 months) was shipped to the external holding facility at 2 weeks rather than 4 weeks postoperatively, a deviation in the postoperative rehabilitation, thus it was excluded from the analysis. Also, one BEAR animal (6 months) had a missing synovial fluid sample, thus was excluded from this study. The total number of animals, analyzed in this study, was 7 in each group at each time point.

After 6 and 12 months, synovial fluid was aspirated from the injured knee joints under anesthesia. For control animals, synovial fluid was obtained under anesthesia within 1 week of arrival to the animal care facility. Samples were centrifuged at 3,000g at room temperature for 10 min and the supernatant stored at −80°C. Following aspiration, animals were euthanized and the knees were harvested and stored at -20°C.

### Proteomics analysis

#### Sample preparation

A multiplexed Isobaric Tag for Relative and Absolute Quantitation (iTRAQ) [18] coupled with liquid chromatography with tandem mass spectrometry (LC-MS/MS) was used to identify the changes in synovial fluid protein levels. iTRAQ is a chemical labeling method, which uses multiplexed isobaric tagging reagent to label peptide mixtures which allows post label mixing of the samples (after enzymatic digestion) without adding complexity to the MS analysis [19]. This will help minimizing the quantitation variability during sample preparation prior to LC MS/MS data collection. The iTRAQ-based quantitative proteomics approach has been widely used to identify biomarkers for several diseases including end stage OA and rheumatoid arthritis [7, 8, 11].

Comparison of the peak areas and resultant peak ratios for MS/MS reporter ions was used to measure the relative abundance of proteins. Synovial fluid from each individual sample was reduced with DTT, alkylated with iodoacetamide, and digested with trypsin at 37°C overnight. Digested samples were separated across five different sets for comparison with each set consisting of seven unique samples (S1 Table). A pooled sample consisting of equal amount of proteins from all 35 total samples was made to serve as a common eighth sample across the five different sets of comparison. Per each comparison set, equal amounts of total peptides were aliquoted and labeled with the 8-plex iTRAQ reagents (iTRAQ Reagents Multiplex kit, Applied Biosystems, CA), then pooled, dried and stored until analyzed by mass spectrometry. Total digested peptides in each sample were quantified by Amino Acid Analysis (AAA; Hitachi Model L-8900) prior to iTRAQ labeling to ensure equal amounts of total peptides for each sample.

#### LC-MS/MS analysis

LC-MS/MS analysis of the iTRAQ labeled samples peptides were carried out using AB SCIEX TripleTOF 5600 mass spectrometer (SCIEX, Framingham, MA) coupled to a NanoACQUITY UPLC system (Waters Inc., Milford, MA). The dried peptide samples were dissolved in 70% formic acid/water, diluted with 13 μl 0.1% trifluoroacetic acid (TFA) and quantitated at A280 absorption on a Nanodrop (ThermoFisher Scientific, Wilmington, DE). Peptides were loaded on a NanoACQUITY Symmetry C18 UPLC Trap column, washed in 9% Buffer A (0.1% formic acid in water) then run for 160 minutes over a linear gradient 99%A to 65% A at 500 nL/min. The parameters utilized on the 5600 Triple-TOF were: 2300 V IonSpray Voltage Floating, Ion Source Gas 1 of 10, Curtain Gas of 20, Interface Heater Temperature 120, with a declustering potential of 60. MS cycle time was 250 milliseconds, with a maximum 20 MS/MS spectra taken each with a 100-millisecond accumulation time, and collision energy adjusted for iTRAQ reagents.

#### Protein abundance level calculation and analysis

The ProteinPilot peptide summary was filtered to only include peptides with zero mis-cleavages, high confidence peptide identifications, and two or more iTRAQ reporter ion ratios measured. We then performed a cyclic Lowess normalization [20] on the remaining peptides to compensate for any differences between iTRAQ labels. All peptides across the 5 sets were merged and normalized to the common pooled sample. This was done by subtracting the common pooled sample, replicated in each set, from the other 7 samples in each set. The purpose of this adjustment is to remove set-specific effects to facilitate comparisons between the experimental groups (i.e. Intact, ACLT and BEAR) and time points (i.e. 6 and 12 months) across all the sets. A linear mixed effect model was used to compare the normalized protein abundance levels between the groups at each time point (limma package in R v3.3.1, The R Foundation for Statistical Computing) [21]. Benjamini and Hochberg method was used to adjust the p-values to control the false discovery rate [22]. P≤0.05 considered statistically significant and used to select differentially abundant proteins between the groups (i.e. Intact, ACLT 6 months, ACLT 12 months, BEAR 6 months and BEAR 12 months). Bivariate linear regression analyses were used to assess the correlations between the macroscopic cartilage damage (total lesion area assessed at 6 or 12 months after ACLT and BEAR) and normalized abundance levels of each identified secreted proteins.

#### Pathway enrichment analysis

The differentially abundant proteins were used to analyze the enrichment of specific pathways using the Process Networks ontology in the MetaCore bioinformatics suite (Thomson Reuters) [23]. Sus scrufa Ensembl identifiers and, if not recognized, gene symbols of the corresponding gene for each protein were used for as input for the analysis. Pathways were considered significantly enriched when they contain at least 2 unique proteins and adjusted P values (false discovery rate; FDR) were less than 0.05.

## Results

**Fig 1:**
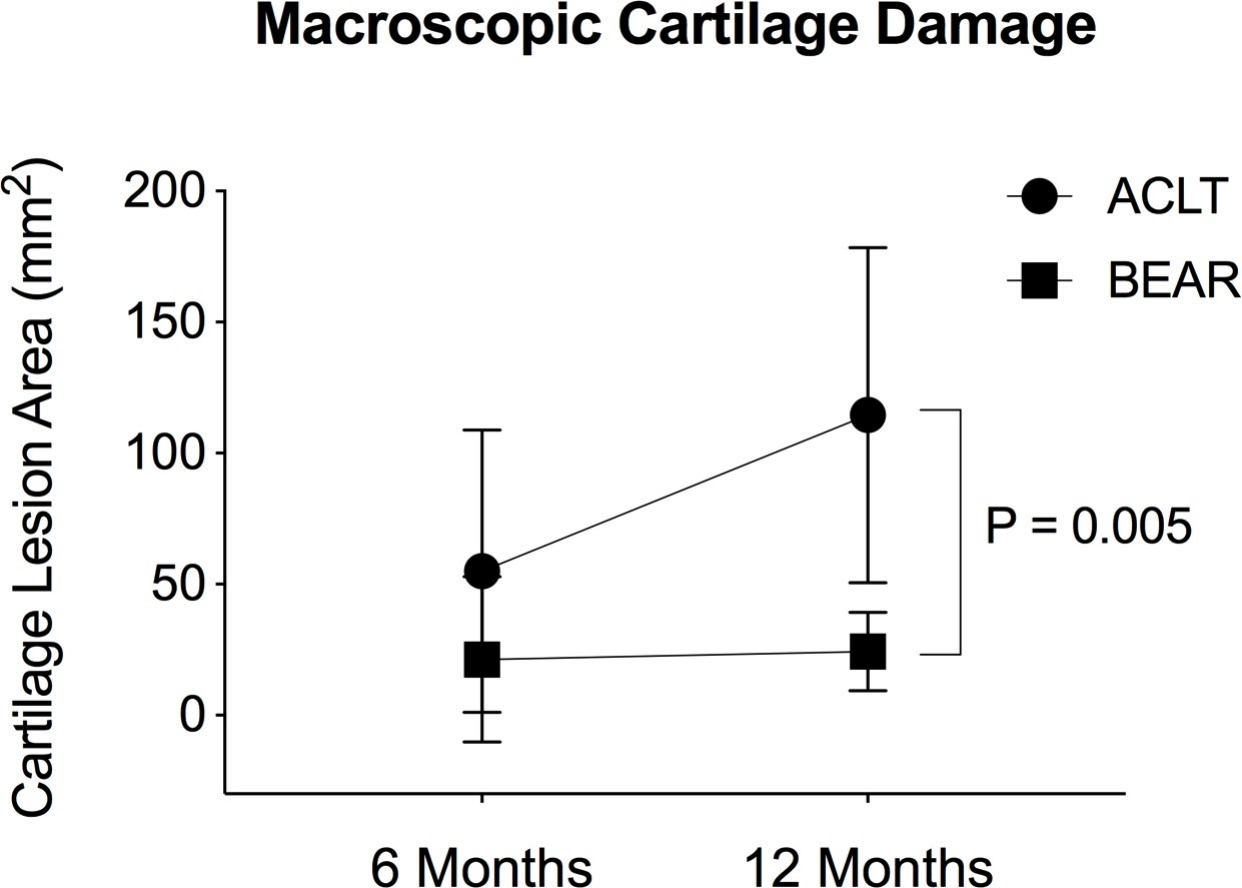
Development of macroscopic cartilage damage following ACL transection. Total cartilage lesion area at 6 and 12 months after untreated ACL injury (ACLT) and bridge-enhanced ACL repair (BEAR). Data is presented as Mean ± SD. P value is derived from a one-way ANOVA with posthoc Bonferroni correction for multiple comparisons (adopted and modified with permission from Murray MM and Fleming BC, Am J Sports Med 2013 [12]) Macroscopic cartilage damage was assessed by measuring the cartilage lesions across the femoral condyles and tibial plateau in medial and lateral compartments using India ink staining and calipers [12].

As previously reported, macroscopic cartilage damage was observed in the ACLT knees, primarily across the medial femoral condyle [12]. While at 6 months no difference in total cartilage lesion area was observed between ACLT and BEAR knees, ACLT knees had a significantly larger total lesion area at 12 months compared to the BEAR knees (Fig 1) [12].

### Most abundant secreted proteins found in the synovial fluid after ACL transection

A total of 1,340 unique peptides corresponding to 232 proteins were identified across all 34 samples (S2 Table). Of the 232 detected proteins, 80 (34%) were secreted extracellular proteins. The subcellular localization patterns of the identified proteins were similar to those reported in knee synovial fluid proteins of humans with knee OA [24]. The top 20 most abundant secreted proteins in the synovial fluid of each surgical group are listed in Table 1. Of the 232 detected proteins, 65 (28%) were significantly differentially abundant in at least one of the comparisons between the experimental groups (i.e. ACLT, BEAR and Intact) and / or between the time points (S3 Table), and 33 (51%) of these were secreted extracellular proteins.

**Table 1:** Top 20 most abundant secreted proteins detected in the synovial fluid in each surgical group. The uniquely abundant proteins in each group are highlighted in bold.

**Abundant Proteins in the ACLT Group at 6 Months**					
**Rank**	**Protein**	**Gene**	**Rank**	**Protein**	**Gene**
1	Peptidylprolyl isomerase A	*PPIA*	11	Serotransferrin	*TF*
2	Vitamin K-dependent protein C	*PROC*	12	**Aggrecan core protein**	***ACAN***
3	Lactadherin	*MFGE8*	13	Corticosteroid-binding globulin	*SERPINA6*
4	Cartilage intermediate layer protein 1	*CILP*	14	Alpha-2-HS-glycoprotein	*AHSG*
5	Coagulation Factor 12	*F12*	15	Protein AMBP	*AMBP*
6	Glucose-6-phosphate isomerase	*GPI*	16	Hemopexin	*HPX*
7	Alpha-1-antitrypsin	*SERPINA1*	17	Complement component C7	*C7*
8	**Pituitary adenylate cyclase-activating polypeptide**	***ADCYAP1***	18	Serum albumin	*ALB*
9	**Haptoglobin**	***HP***	19	Apolipoprotein E	*APOE*
10	Inhibitor of carbonic anhydrase	*ICA*	20	Clusterin	*CLU*
**Abundant Proteins in the BEAR Group at 6 Months**					
**Rank**	**Protein**	***Gene***	**Rank**	**Protein**	**Gene**
1	Peptidylprolyl isomerase A	*PPIA*	11	Serotransferrin	*TF*
2	Vitamin K-dependent protein C	*PROC*	12	Complement component C7	*C7*
3	**Plasminogen activator inhibitor 1**	***SERPINE1***	13	**Apolipoprotein C3**	***APOC3***
4	Cartilage intermediate layer protein 1	*CILP*	14	Apolipoprotein E	*APOE*
5	Lactadherin	*MFGE8*	15	Inhibitor of carbonic anhydrase	*ICA*
6	Glucose-6-phosphate isomerase	*GPI*	16	Serum albumin	*ALB*
7	Coagulation Factor 12	*F12*	17	Protein AMBP	*AMBP*
8	Corticosteroid-binding globulin	*SERPINA6*	18	Hemopexin	*HPX*
9	Alpha-1-antitrypsin	*SERPINA1*	19	Alpha-2-HS-glycoprotein	*AHSG*
10	**Elafin**	***PI3***	20	Clusterin	*CLU*
**Abundant Proteins in the ACLT Group at 12 Months**					
**Rank**	**Protein**	**Gene**	**Rank**	**Protein**	**Gene**
1	Cystatin-B	*CSTB*	11	Hyaluronan and proteoglycan link protein 1	*HAPLN1*
2	Protein S100-A12	*S100A12*	12	**Peptidylprolyl isomerase A**	***PPIA***
3	Complement C5	*C5*	13	Aggrecan core protein	*ACAN*
4	Lactotransferrin	*LTF*	14	Alpha-1-antitrypsin	*SERPINA1*
5	von Willebrand factor	*VWF*	15	Cathepsin D	*CTSD*
6	**Vitamin K-dependent protein C**	***PROC***	16	**Prothrombin**	***F2***
7	**Prophenin-2**		17	Complement factor D	*CFD*
8	Insulin-like growth factor-binding protein 2	*IGFBP2*	18	Prophenin-1	
9	Complement C1q subcomponent subunit A	*C1QA*	19	**Decorin**	***DCN***
10	**Chitinase-3-like protein 1**	***CHI3L1***	20	**Thyroxine-binding globulin**	***SERPINA7***
**Abundant Proteins in the BEAR Group at 12 Months**					
**Rank**	**Protein**	**Gene**	**Rank**	**Protein**	**Gene**
1	Hyaluronan and proteoglycan link protein 1	*HAPLN1*	11	Protein S100-A12	*S100A12*
2	Cystatin-B	*CSTB*	12	Complement C1q subcomponent subunit A	*C1QA*
3	**Cartilage intermediate layer protein 1**	***CILP***	13	Complement factor D	*CFD*
4	von Willebrand factor	*VWF*	14	**Apolipoprotein A4**	***APOA4***
5	**Annexin A2**	***ANXA2***	15	Insulin-like growth factor-binding protein 2	*IGFBP2*
6	Complement C5	*C5*	16	**Clusterin**	***CLU***
7	Cathepsin D	*CTSD*	17	**Alpha-2-HS-glycoprotein**	***AHSG***
8	**Apolipoprotein M**	***APOM***	18	Prophenin-1	
9	**Annexin A1**	***ANXA1***	19	Alpha-1-antitrypsin	*SERPINA1*
10	Aggrecan core protein	*ACAN*	20	Lactotransferrin	*LTF*

### Treatment related changes in synovial fluid proteome

At 6 months, a total of 27 secreted proteins were significantly differentially abundant between intact group and surgical groups (ACLT and/or BEAR; Table 2). There were no significant differences in the normalized abundance levels of the detected secreted proteins between the ACLT and BEAR groups at 6 months.

**Table 2:** Secreted proteins that are significantly different in abundance in ACLT and/or BEAR knees compared to intact group at 6 months. Fold change values represent the ratio of detection in the ACLT or BEAR group versus intact group; thus a fold change greater than 1 reflects higher protein abundance in the synovial fluid of the ACLT or BEAR group and a fold change less than 1 reflects higher protein abundance in the synovial fluid of the intact group.

Protein	Gene	ACLT / Intact		BEAR / Intact	
		Fold Change	*P*-Value	Fold Change	*P*-Value
**Differentially Abundant Proteins in both ACLT and BEAR Groups Compared to Intact**					
Vitamin K-dependent protein C	*PROC*	6	1.03E-06	7.35	6.91E-08
Cartilage intermediate layer protein 1	*CILP*	3.82	5.51E-03	5.35	8.34E-04
Coagulation Factor XII	*F12*	2.71	2.74E-04	2.67	3.34E-04
Alpha-1-antitrypsin	*SERPINA1*	1.62	8.92E-04	1.56	1.80E-03
Clusterin	*CLU*	1.6	6.52E-05	1.65	2.66E-05
Inhibitor of carbonic anhydrase	*ICA*	1.6	1.02E-04	1.47	8.87E-04
Hemopexin	*HPX*	1.59	2.16E-04	1.52	6.56E-04
Serotransferrin	*TF*	1.42	6.18E-05	1.5	7.23E-06
Apolipoprotein A4	*APOA4*	1.38	2.59E-02	1.63	1.22E-03
Protein AMBP	*AMBP*	1.37	1.23E-02	1.31	2.99E-02
Serum albumin	*ALB*	1.13	3.68E-02	1.18	4.55E-03
Transthyretin	*TTR*	0.83	2.77E-02	0.79	8.35E-03
Inter-α trypsin inhibitor heavy chain 2	*ITIH2*	0.8	1.99E-02	0.76	5.95E-03
Complement factor B	*CFB*	0.69	3.64E-02	0.72	4.43E-02
Ig lambda-1 chain C regions	*IGLC1*	0.59	2.36E-03	0.59	2.16E-03
Complement factor D	*CFD*	0.58	3.05E-04	0.61	1.17E-03
Ficolin-2	*FCN2*	0.39	5.27E-03	0.54	3.98E-02
**Differentially Abundant Proteins in ACLT Compared to Intact**					
Haptoglobin	*HP*	3.21	8.33E-04		
Coagulation factor V	*F5*	0.09	4.33E-02		
**Differentially Abundant Proteins in BEAR Compared to Intact**					
Peptidylprolyl isomerase A	*PPIA*			11.53	1.74E-02
Apolipoprotein C3	*APOC3*			1.75	4.57E-03
Apolipoprotein E	*APOE*			1.37	2.13E-02
Apolipoprotein A1	*APOA1*			1.33	2.08E-02
Prothrombin	*F2*			0.73	1.13E-02
Vitronectin	*VTN*			0.71	5.80E-03

At 12 months, a total of 16 secreted proteins were significantly differentially abundant between intact group and surgical groups (ACLT and/or BEAR; Table 3). Compared to the BEAR group, the ACLT group had higher normalized abundance levels of vitamin K-dependent protein C (by 2.4 fold, P = 0.00126) along with lower normalized abundance levels of apolipoprotein A4 (by 0.7 fold, P = 0.0213) and cartilage intermediate layer protein (by 0.35 fold, P = 0.049).

**Table 3:** Secreted proteins that are significantly different in abundance in ACLT and/or BEAR knees compared to intact group at 12 months. Fold change values represent the ratio of detection in the ACLT or BEAR group versus intact group; thus a fold change greater than 1 reflects higher protein abundance in the synovial fluid of the ACLT or BEAR group and a fold change less than 1 reflects higher protein abundance in the synovial fluid of the intact group.

Protein	Gene	ACLT / Intact		BEAR / Intact	
		Fold Change	*P*-Value	Fold Change	*P*-Value
**Differentially Abundant Proteins in both ACLT and BEAR Groups Compared to Intact**					
Trypsin-1	*PRSS1*	2.23	1.06E-02	2.24	8.52E-03
Haptoglobin	*HP*	1.96	4.03E-02	1.91	4.32E-02
Actin beta	*ACTB*	1.52	1.58E-02	1.65	3.55E-03
Clusterin	*CLU*	1.43	1.28E-03	1.7	7.33E-06
Apolipoprotein A4	*APOA4*	1.36	3.17E-02	1.86	5.79E-05
Apolipoprotein A1	*APOA1*	1.29	3.71E-02	1.43	3.90E-03
Vitronectin	*VTN*	0.77	3.28E-02	0.64	4.63E-04
Inter-α trypsin inhibitor heavy chain family member 4	*ITIH4*	0.63	6.81E-03	0.55	6.32E-04
**Differentially Abundant Proteins in ACLT Compared to Intact**					
Serotransferrin	*TF*	1.2	2.14E-02		
**Differentially Abundant Proteins in BEAR Compared to Intact**					
Cartilage intermediate layer protein	*CILP*			2.83	5.01E-02
Annexin A1	*ANXA1*			2.48	4.36E-02
Plasminogen	*PLG*			0.84	4.88E-02
Inter-α trypsin inhibitor heavy chain 1	*ITIH1*			0.8	1.69E-02
Inter-α trypsin inhibitor heavy chain 2	*ITIH2*			0.8	1.67E-02
Ig lambda-1 chain C regions	*IGLC1*			0.68	1.73E-02
Vitamin K-dependent protein C	*PROC*			0.44	1.99E-03

There were 20 secreted synovial fluid proteins with significant fold changes between the 6 to 12-month time points in ACLT and/or BEAR knees (Table 4). Nine proteins had significant fold changes from 6 to 12 months after surgery in both groups. Five proteins had a significant fold change from 6 to 12 months only after untreated ACL transection. Six proteins had a significant fold change from 6 to 12 months only after bridge-enhanced ACL repair.

**Table 4:** Secreted proteins that are significantly different in abundance between 6 and 12 months after surgery.

**Differentially abundant in both ACLT and BEAR**					
**Protein**	**Gene**	**ACLT (12 / 6 months)**		**BEAR (12 / 6 months)**	
		**Fold Change**	***P*-Value**	**Fold Change**	***P*-Value**
**Increased from 6 months to 12 months**					
Complement factor D	*CFD*	1.80	8.97E-05	1.81	4.98E-05
Transthyretin	*TTR*	1.29	3.36E-03	1.37	2.69E-04
**Decreased from 6 months to 12 months**					
Serotransferrin	*TF*	0.85	2.93E-02	0.72	5.78E-05
Serum albumin	*ALB*	0.85	4.13E-03	0.76	1.05E-05
Inhibitor of carbonic anhydrase	*ICA*	0.74	5.26E-03	0.77	1.09E-02
Apolipoprotein E	*APOE*	0.73	1.61E-02	0.68	3.14E-03
Protein AMBP	*AMBP*	0.73	9.04E-03	0.69	2.07E-03
Coagulation Factor 12	*F12*	0.60	3.46E-02	0.55	1.18E-02
Vitamin K-dependent Protein C	*PROC*	0.17	1.47E-06	0.06	8.04E-10
**Differentially abundant only in ACLT**					
**Protein**	**Gene**	**ACLT (12 / 6 months)**			
		**Fold Change**	***P*-Value**		
**Increased from 6 months to 12 months**					
Insulin-like growth factor-binding protein 2	*IGFBP2*	1.80	2.63E-02		
Ig lambda-1 chain C regions	*IGLC1*	1.45	2.20E-02		
Thyroxine-binding globulin	*SERPINA7*	1.45	1.24E-02		
**Decreased from 6 months to 12 months**					
Hemopexin	*HPX*	0.72	4.98E-03		
Cartilage intermediate layer protein 1	*CILP*	0.26	5.46E-03		
**Differentially abundant only in BEAR**					
**Protein**	**Gene**			**BEAR (12 / 6 months)**	
				**Fold Change**	***P*-Value**
**Increased from 6 months to 12 months**					
Annexin A1	*ANXA1*			3.82	2.81E-03
**Decreased from 6 months to 12 months**					
Alpha-1-antitrypsin	*SERPINA1*			0.73	1.51E-02
Complement component C7	*C7*			0.58	2.73E-02
Apolipoprotein C3	*APOC3*			0.56	1.87E-03
Leukocyte elastase inhibitor	*SERPINB1*			0.42	3.84E-02
Peptidylprolyl isomerase A	*PPIA*			0.07	6.66E-03

Differentially abundant proteins enriched 7 biological pathways (Process Network ontology) including terms related to blood coagulation, proteolysis, inflammation and cell adhesion (Table 5).

**Table 5:** Biological pathways enriched by differentially abundant proteins at 6 months.

Pathway	FDR	Proteins
Blood Coagulation	1.18E-04	Vitamin K-dependent protein C, Prothrombin, Plasminogen, Coagulation Factor V, Coagulation Factor XII, Alpha-1-antitrypsin
Proteolysis (ECM Remodeling)	3.50E-04	Plasminogen, Prothrombin, Vitronectin, Coagulation Factor XII, Trypsin I, Clusterin, Alpha-1-antitrypsin
Inflammation (Kallikrein-Kinin System)	7.63E-04	Inter-α trypsin inhibitor heavy chain family member 4, Prothrombin, Plasminogen, Coagulation Factor V, Coagulation Factor XII, Alpha-1-antitrypsin
Inflammation (Protein C Signaling)	7.63E-04	Vitamin K-dependent protein C, Actin Beta, Prothrombin, Plasminogen, Coagulation Factor V
Inflammation (Complement System)	7.63E-04	Complement Component C7, Ficolin, Clusterin, Complement Factor B, Complement Factor D
Cell Adhesion (Platelet-Endothelium-Leucocyte Interactions)	1.79E-03	Vitamin K-dependent protein C, Prothrombin, Plasminogen, Coagulation Factor V, Vitronectin, Coagulation Factor XII
Proteolysis (Connective Tissue Degradation)	4.51E-02	Plasminogen, Vitronectin, Trypsin 1, Alpha-1-antitrypsin

### Associations between protein levels and total area of macroscopic articular cartilage damage

**Fig 2:**
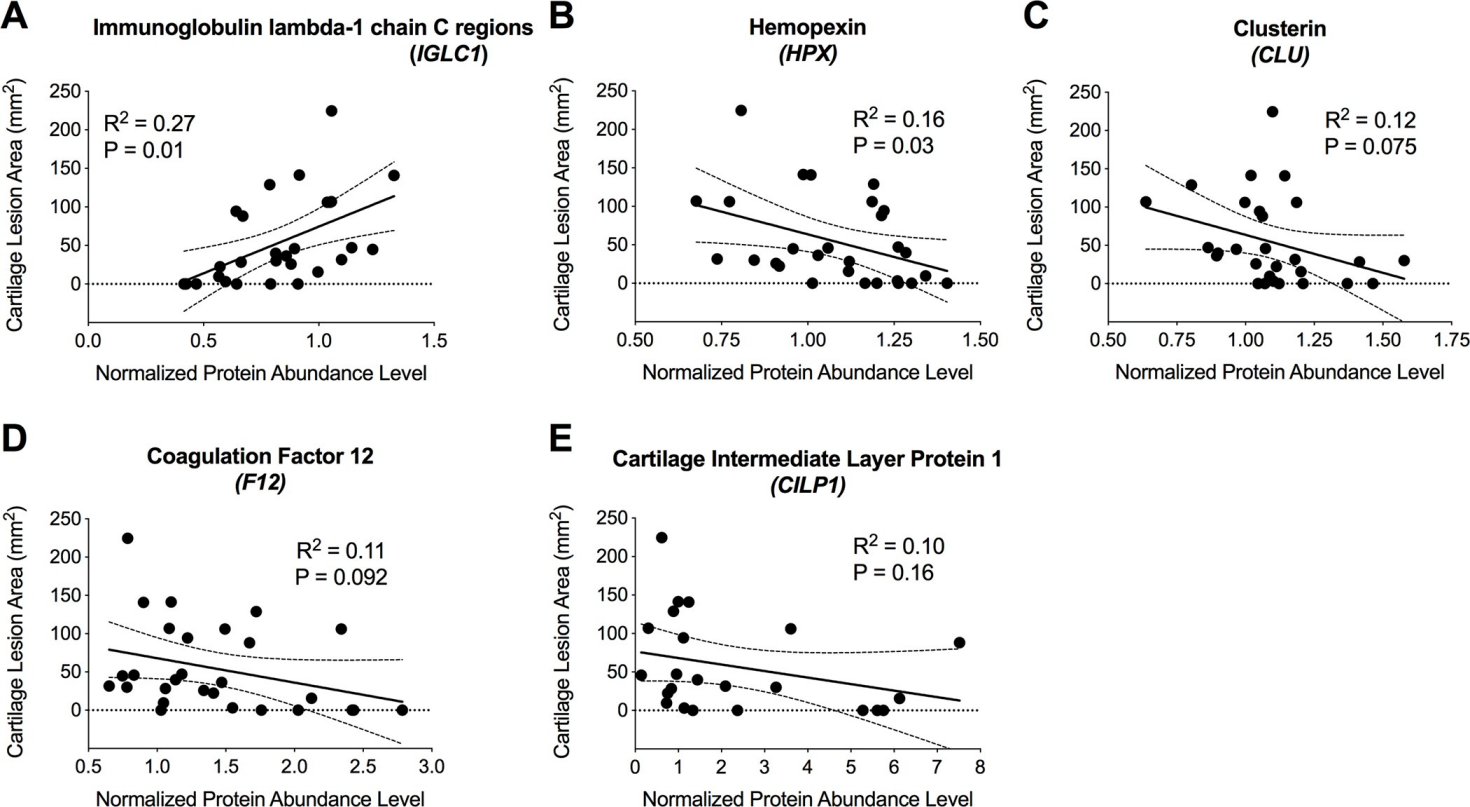
Associations between macroscopic cartilage damage and secreted proteins concentrations. Correlations between macroscopic cartilage damage area and the normalized abundance levels of (A) Ig lambda-1 chain C regions, (B) Hemopexin, (C) Clusterin, (D) Coagulation factor 12 and (E) Cartilage intermediate layer protein 1 for both ACLT and BEAR groups at 6 and 12 months.

Among all the identified secreted proteins, notable associations (R^2^ ≥ 0.1) were only found between the total area of the macroscopic cartilage damage across the femoral condyles and tibial plateau and the normalized abundance levels of Ig lambda-1 chain C regions (r = 0.52), Hemopexin (r = -0.40), Clusterin (r = -0.35), Coagulation factor 12 (r = -0.32) and Cartilage intermediate layer protein 1 (r = -0.33); Fig 2.

## Discussion

The results of this study support our hypothesis that development of macroscopic cartilage damage following an ACL injury and subsequent surgery are accompanied with changes in synovial fluid proteome with different responses in untreated knees compared to repaired joints. There was no significant difference in cartilage lesion area or quantitative protein abundance levels between the two treatment groups at 6 months. However, by 12 months after surgery, there was significantly greater cartilage damage in the knees with an untreated ACL transection than in those knees that underwent bridge-enhanced ACL repair (Fig 1). This damage was accompanied by differences in the proteome between the groups at 12 months, with the ACLT group having a higher concentration of Vitamin K-dependent Protein C (2.4 fold), and a decreased content of Apolipoprotein A4 (0.7 fold) and Cartilage intermediate layer protein 1 (0.35 fold) when compared to the BEAR knees. In addition, there was also a difference in the proteins which significantly changed in abundance between 6 and 12 months (Table 4), with Insulin-like growth factor-binding protein 2 (IGFBP2), Ig lambda-1 chain C regions (IGLC1), Hemopexin and CILP changing in the ACLT group, and significant changes in Annexin A1, alpha-1 antitrypsin, Complement C7, Apolipoprotein C3, Leukocyte elastase inhibitor and Peptidylprolyl isomerase A (PPIA) found in the BEAR group. Increased levels of IGLC1 and decreased levels of Hemopexin, Clusterin, Coagulation factor 12 and CILP were also found to correlate with greater cartilage lesion area when the knees from both groups were pooled (Fig 2).

While there were no statistically significant differences in protein abundance between the surgical groups at 6 months after surgery, the makeup of the synovial fluid proteome, and specifically the top 20 most abundant proteins in the fluid, were similar but not identical in the two surgical groups. Seventeen of the 20 most abundant secreted proteins (Table 1) were the same in ACLT and BEAR groups, 15 of which have been previously reported to be abundant in synovial fluid of human knees with OA [8, 11, 24]. For the six differences (three in each group) in the top 20 secreted proteins, the ACLT knees had 2 additional pro-inflammatory proteins (Haptoglobin and Pituitary adenylate cyclase-activating polypeptide) while the BEAR group had two protease inhibitors at 6 months (Plasminogen activator inhibitor 1 and Elafin). The pro-inflammatory role of Haptoglobin, Pituitary adenylate cyclase-activating polypeptide (PACAP), Plasminogen activation and Elastase are well documented [25–28]. High synovial fluid concentrations of Haptoglobin, Plasminogen (or Plasmin) and Elastase have been reported in arthritic knees [11, 24, 29–31]. PACAP-deficiency in mice has shown to result in lower inflammation in early arthritis followed by increased immune cell function and bone formation (e.g. osteophytes) in late arthritis [32]. The presence of Haptoglobin and PACAP among the most abundant proteins in the ACLT group in addition to the presence of Plasminogen activator inhibitor 1 and Elafin among the most abundant proteins in the BEAR group at 6 months might be indicative of a more pro-inflammatory environment in ACLT knees at early stages of the disease. Further studies would be needed to validate this hypothesis.

In addition to proteases and protease inhibitors, Apolipoprotein C3 and Aggrecan core protein (Aggrecan) were the other two secreted proteins which were uniquely present in the BEAR or ACLT top 20 abundance lists, respectively, at 6 months (Table 1). Apolipoprotein C3 has previously been identified in the synovial fluid of human knees with OA [24], and its plasma levels have been found to be elevated in patients with non-progressing radiographic knee OA compared to those who progress [33]. This is reflected by our finding of Apolipoprotein C3 among the most abundant proteins in the non-progressive BEAR group and not in the progressive ACLT group at 6 months (Table 1). Aggrecan has also been previously detected in synovial fluid of knees with OA [11, 24]; however, in this case, increased Aggrecan in the synovial fluid has been associated with greater cartilage damage in osteoarthritic joints [34–37]. This is reflected in our study where Aggrecan was among the most abundant proteins in the ACLT group and not in BEAR or intact groups (Table 1).

At 12 months after surgery, there were significant differences in protein abundance levels between the BEAR and ACLT groups, with an increase in Vitamin K-dependent protein C and decreases in Apolipoprotein A4 and CILP seen in the ACLT group. Vitamin K-dependent protein C is an activator of several matrix metalloproteinases (MMPs), including MMP2, 9 and 13, and has shown to be significantly elevated in the synovium and synovial fluid of patients with rheumatoid arthritis (RA) and OA [38]. While Apolipoprotein A4 has been detected in the synovial fluid of human knees with OA [8, 24], their role in OA pathogenesis is not well understood and warrants further studies. Considering recent reports on the role of lipid metabolism in the OA development [39, 40], it is possible that apolipoprotein proteins affects knee OA progression by regulating the lipid metabolism and efflux. CILP is a large secreted glycoprotein with major role in cartilage scaffolding and new cartilage formation [41]. Increased CILP synthesis by chondrocytes has also been reported in early-stage OA [42, 43]. which can be indicative of joint response to repair damaged cartilage [44]. The consistently higher abundance of CILP in BEAR knees compared to intact (at 6 and 12 months) and ACLT (at 12 months) knees may suggest a more chondroprotective environment in repaired knees (Tables 2 & 3).

In addition, at 12 months, the top 20 most abundant proteins in the ACLT knees had 5 additional pro-inflammatory proteins (Vitamin K-dependent protein C, Prothrombin, PPIA and Chitinase-3-like protein 1) and 3 fewer anti-inflammatory proteins (Clusterin, Alpha-2-HS glycoprotein and Annexin A1; Table 1). The pro-inflammatory role of Vitamin K-dependent protein C, Prothrombin, PPIA and Chitinase-3-like protein 1, as well as anti-inflammatory role of Clusterin, Alpha-2-HS glycoprotein (Fetuin-A) and Annexin A1 in arthritic joints are well documented [25–28, 45–61]. Elevated levels of these pro-inflammatory proteins have been reported in serum and synovial fluid of patients with RA and OA [8, 24, 49, 62–65]. Prophenin-2 is a porcine specific Cathelicidin antimicrobial peptide, with similar pro-inflammatory properties as LL-37 (the only identified Cathelicidin antimicrobial peptide in humans), which is regulated by macrophages, polymorphonuclear leukocytes, and keratinocytes [66]. A recent study has shown strong overexpression of Cathelicidin in joints of patients with RA and rats with pristane-induced arthritis [67]. A recent proteomic analysis has also reported LL-37 among identified synovial fluid proteins in the patients with knee OA [24]. The presence of pro-inflammatory proteins among the most abundant proteins in the ACLT group and the anti-inflammatory proteins among the most abundant proteins in the BEAR group at 12 months suggests there may be a greater pro-inflammatory environment in ACLT knees at a later stage of the disease. This is further supported by the observed significantly lower abundance of the Vitamin K-dependent protein C in BEAR knees compared to ACLT and intact groups as well as higher abundance of Annexin A1 in the BEAR knees compared to intact group.

In comparing the proteomes at 6 and 12 months after surgery (Table 4), there were significant increases in IGFBP2 and IGLC1 levels, as well as significant decreases in CILP and Hemopexin levels in the synovial fluid of ACLT knees from 6 to 12 months. IGFP2 is synthesized by synoviocytes and chondrocytes and regulates Insulin-like growth factor (IGF) [68, 69], a key promoter of cartilage growth [70, 71]. Increased levels of IGFBPs have been reported in the synovial fluid of arthritic joints [72, 73], and serum concentration has been correlated with RA disease severity [74–76]. Hemopexin has a negative modulatory function on T-cell differentiation [77, 78], and exogenous administration of Hemopexin has recently been shown to result in increased glycosaminoglycan deposition [79]. In contrast to increased synovial concentrations of pro-inflammatory proteins (IGFBP2 and IGLC1) in the ACLT knees, there were significant decreases in the synovial concentrations of two pro-inflammatory proteins (PPIA and complement C7) in the BEAR knees from 6 to 12 months.

Biological pathways enriched by the changes in synovial fluid protein content were related to blood coagulation, proteolysis, inflammation and cell adhesion (Table 5). Among those, proteolysis (ECM remodeling) and inflammation (complement pathway) pathways have consistently shown to play an active role in OA pathophysiology [8, 80–83]. The role of remaining 5 enriched pathways in the development of OA is less studied. Recent comparative analysis of synovial fluid in patients with healthy knees with those with knee OA (both early and late stages) have shown significant enrichment of blood coagulation pathway by differentially abundant proteins between the groups [8]. Our group has also shown enrichment of cell adhesion (platelet-endothelium-leucocyte interactions) and proteolysis (connective tissue degradation) pathways by differentially expressed genes in the synovium and cartilage within 2 weeks after ACL injury in a minipig model of posttraumatic OA [80, 81]. These findings, in addition to abovementioned evidence supporting the involvement of the proteins / genes, which enriched these pathways, in OA pathophysiology underscore their importance as potential targets to mediate the biologic joint response to injury in an effort to lower the risk of posttraumatic OA.

### Limitations

Unbiased LC MS/MS proteomics techniques are primarily limited to detection of more highly abundant proteins within a complex biological matrix. Antibody-based depletion methods could have been used to improved our ability to quantify lower abundance level proteins by removing the top abundance level proteins. However, they can also result in removal of lower abundance level proteins and ultimately pose challenges in accurate quantification of protein contents [84]. In our discovery workflow, we wanted to capture a “global” representation of proteins in the synovial fluid, as such chose to avoid antibody-based depletion. To better compensate and delve deeper into the synovial fluid proteome, additional in-depth and/or targeted approaches are required to further study less abundant proteins that are likely present but not detectable or quantifiable using this technique (i.e. Lubricin, matrix metalloproteinases and cytokines). We have previously used similar MS-based proteomics approach to study the synovial fluid proteome of healthy porcine knees [15]. The substantial overlap between those findings and identified proteins here is reassuring and supports our MS-based protein discovery approach. However, further efforts are essential for orthogonal validation of the identified proteins in individual samples compared to the pooled samples in independent cohorts. Another limitation is the cross-sectional nature of the study. While the cross-sectional time points have enabled us to macroscopically assess the cartilage health, longitudinal analyses are required to better characterize the changes in synovial fluid proteome in response to joint degeneration over time. Finally, the current results only support associations between cartilage damage and certain synovial fluid proteins in early posttraumatic OA. Further functional studies are required to investigate the mechanistic nature of these associations and to determine the potential application of suggested targets as biomarkers for OA progression or as disease modifiers for development of novel therapeutics.

## Conclusions

Knees treated with a surgical procedure that results in posttraumatic OA (ACLT group) had higher concentrations of pro-inflammatory proteins and lower concentrations of anti-inflammatory proteins than knees treated with a surgical procedure that does not result in post-traumatic OA (bridge-enhanced ACL repair). In addition, the group developing posttraumatic OA had a lower and declining synovial concentrations of CILP, in contrast to a consistently high abundance of CILP in repaired knees. These differences suggest that the knees treated with bridge-enhanced ACL repair may be maintaining an environment that is more protective of the extracellular matrix, a function which is not seen in the ACLT knees.

## Supporting information

S1 Table iTRAQ labeling of the synovial fluid samples. (XLSX)

S2 Table Detected synovial fluid proteins. (XLSX)

S3 Table Significant differentially abundant proteins between groups. (XLSX)

S1 File ARRIVE guidelines checklist. (PDF)
